# The PD COMM Process Evaluation: Describing Interventions and Implementation in a UK Pragmatic Randomised Controlled Trial of Speech and Language Therapy for People With Parkinson's‐Related Dysarthria

**DOI:** 10.1111/1460-6984.70084

**Published:** 2025-07-10

**Authors:** Avril Nicoll, Marian C. Brady, Patricia Masterson‐Algar, Christopher Burton, Gillian Beaton, Sylvia Dickson, Maria Caulfield, Christina H. Smith, Carl E. Clarke, Natalie Ives, Sue Jowett, Caroline Rick, Rebecca Woolley, Catherine M. Sackley, on behalf of the PD COMM collaborative group

**Affiliations:** ^1^ Aberdeen Centre for Women's Health Research University of Aberdeen Aberdeen UK; ^2^ School of Health and Life Sciences Glasgow Caledonian University Glasgow UK; ^3^ School of Health Sciences Bangor University Bangor UK; ^4^ School of Health Sciences University of East Anglia Norwich UK; ^5^ NHS Greater Glasgow and Clyde, SLT Department New Victoria Hospital Glasgow UK; ^6^ Centre for Applied Dementia Studies, Faculty of Health Studies University of Bradford Bradford West Yorkshire UK; ^7^ NHS Lothian, Speech & Language Therapy Department Department of Clinical Neurosciences Edinburgh UK; ^8^ Department of Applied Health Sciences University of Birmingham Birmingham UK; ^9^ Birmingham Clinical Trials Unit, College of Medicine and Health University of Birmingham Birmingham UK; ^10^ Nottingham Clinical Trials Unit University of Nottingham Nottingham UK; ^11^ School of Health Science University of Nottingham Nottingham UK

**Keywords:** implementation science, intervention study, Parkinson disease, pragmatic clinical trial, randomized controlled trial, speech and language therapy

## Abstract

**Background:**

As people with Parkinson's experience progressive communication changes, effective, implementable speech and language therapy (SLT) interventions are needed. Process evaluations alongside pragmatic randomised controlled trials (RCTs) are of clinical value if they describe, compare and understand the implementation of trial interventions. This paper reports the PD COMM process evaluation. PD COMM was a large, UK multi‐centre phase III pragmatic RCT of SLT in the National Health Service (NHS). It recruited 388 people with Parkinson's who were randomised to Lee Silverman Voice Treatment (LSVT), Standard NHS SLT, or no dysarthria intervention.

**Aims:**

To describe and compare the content and service delivery components of the PD COMM SLT interventions; understand experiences of implementing LSVT; explain trial outcomes; and reflect on implications for practice and research.

**Methods and Procedures:**

We took a pragmatic, mixed methods approach. The intervention description team used a sub‐sample of routine therapy notes and trial record forms, the Template for Intervention Description and Replication (TIDieR) and simple descriptive statistics to compare Individual Participant Therapy Data (LSVT *n* = 51; Standard NHS SLT *n* = 54). In parallel, informed by Normalisation Process Theory (NPT), the implementation team conducted qualitative interviews with a sub‐sample of therapists (*n* = 20) and participants (*n* = 24) to understand the additional work of implementing LSVT. The core process evaluation team met to integrate the findings in relation to the trial outcomes.

**Outcomes and Results:**

LSVT was largely delivered per protocol, tailored to participants’ interests and interactions. Dosage was a key difference between the two interventions, commonly achieved by two or more therapists delivering LSVT. Effective mechanisms were LSVT's structured design, repetitive and social nature, practise requirements and focus on volume. Standard NHS SLT was eclectic, reflecting a range of clinical approaches at a lower intensity, including some techniques and activities in common with LSVT. Although focused on impairment therapy, including specific voice therapy techniques, it also featured cognitive‐linguistic and psychosocial targets and low technology augmentative and alternative communication (AAC). The trial design may have limited opportunities for group intervention.

**Conclusions and Implications:**

Any LSVT roll‐out needs service support and coordination, and should take an inclusive approach. Future research of Standard NHS SLT should explore a rationale for dosage and more explicit tailoring to individuals and their families. There is also a pressing need to deliver the benefits of LSVT in a cost‐effective manner and to develop a range of evidence‐based, implementable alternatives as people's communication support needs change.

**WHAT THIS PAPER ADDS:**

*What is already known on the subject*

- Lee Silverman Voice Treatment (LSVT) has a body of incrementally‐developed evidence from effectiveness trials but has not previously been tested in a pragmatic randomised controlled trial (RCT) with an embedded process evaluation.

*What this paper adds to the existing knowledge*

- This mixed methods process evaluation paper describes and compares content and service delivery components to understand similarities and differences between LSVT and Standard NHS SLT interventions and experiences of implementing LSVT in the UK NHS.

*What are the potential or actual clinical implications of this work?*

- Services can use the findings to plan delivery of intensive interventions and to reflect on the content and service delivery aspects of locally Standard NHS SLT and how it might be improved.

## 1 Background

People living with Parkinson's experience a variety of progressive communication changes which can profoundly affect how they are perceived and feel about themselves. These include hypokinetic dysarthria (quiet voice, reduced variation in use of intonation and emphasis, imprecise articulation, altered speech rate), cognitive‐linguistic deterioration, and problems managing interactions (Miller 2017). They weigh up the value of speaking against the effort required and the energy needed for other things (Miller et al. 2006, Yorkston et al. 2017b), and reduced participation in conversations is a common experience (Johansson et al. 2020). The communication environment, such as group or noisy settings, has a negative impact (Wylie et al. 2022), as does emotion and fatigue (Schalling et al. 2018). Communication support needs of individuals and families change over the course of the disease (Baylor et al. 2024), as progressive physical disability and cognitive decline impact on independence and place strain on relationships through changing roles and loss of joint activities, intimacy, and a social life (Glover et al. 2023). It is therefore imperative that a range of acceptable, implementable interventions with high level clinical and cost‐effectiveness evidence are available to people with Parkinson's‐related dysarthria, their families and therapists.

There are, however, at least three barriers to accessing this breadth of intervention evidence. These encompass methodological tensions, power imbalances, and inadequate reporting.

*Methodological challenges*: Standardisation and measurement of SLT intervention content, service delivery and related outcomes are challenging because human communication patterns, impairments and needs are complex and usually individualistic. Historically, this fostered an uneasy relationship between the speech and language therapy (SLT) profession and randomised controlled trials (RCTs), which evaluate effectiveness based on average effects at a group level (Carding and Hillman 2001).

*Power imbalances*: The recent literature for Parkinson's‐related dysarthria SLT is dominated by trials of one trademarked intervention, Lee Silverman Voice Treatment (LSVT) (see e.g., Levy et al. 2020) and its variants. Although qualitative research into patient experiences (Gillivan‐Murphy et al. 2019) and co‐production of new complex interventions (Clay et al. 2024) are growing, recent empirical data on patterns of intervention content and service delivery in routine clinical practice is limited (e.g., Swales et al. 2019), and there is a dearth of studies focused on implementation.

**FIGURE 1:**
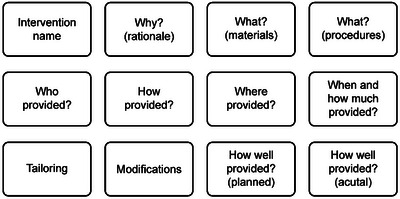
TIDieR categories.

*Inadequate reporting*: Standardised reporting of complex interventions is a prerequisite to the transparency needed for robust evaluation, comparison and implementation. The Template for Intervention Description and Replication (TIDieR) checklist (Hoffmann et al. 2014) (Figure 1) was developed to encourage description of interventions in clinical trials to a replicable standard, but in 2017 only 28% (*n* = 46) of the 162 interventions in recent RCTs of SLT met the TIDieR benchmark (Ludemann et al. 2017).

Pragmatic trials offer opportunities to address these barriers, as they ask if interventions work under usual conditions in the real world of clinical practice (Loudon et al. 2015). Process evaluations perform a vital role in explaining trial outcomes by, for example, describing the interventions, the trial, the pre‐existing context, implementation, and potential ‘mechanisms’ of impact (Moore et al. 2015). To be of clinical value, descriptions of ‘usual care’ in pragmatic RCTs are as important as descriptions of experimental interventions (Nicholls et al. 2020). A systematic review of process evaluations in pragmatic RCTs (*n* = 31) confirmed they are a rich source of knowledge, but are currently suboptimal, as they vary considerably in nature and are often inadequately labelled and reported (French et al. 2020).

PD COMM was a large, UK multi‐centre phase III pragmatic RCT of SLT in the UK National Health Service (NHS). It recruited 388 people with Parkinson's with reported speech or voice problems from September 2016 to March 2020 (Sackley et al. 2024b). The trial was registered (ISRCTN12421382) and was approved by the West Midlands—Coventry and Warwickshire Research Ethics Committee (15/WM/0443). Trial design was informed by a pilot (Sackley et al. 2018); this included selecting the patient‐reported Voice Handicap Index as the primary outcome measure to capture the interventions’ impact on functional communication. Participants were randomised to one of two SLT intervention groups—Lee Silverman Voice Treatment (LSVT LOUD, referred to here as LSVT) or Standard NHS SLT (referred to here as SNHS SLT)—or to a no dysarthria intervention control group. Depending on local practice, any of these groups could constitute ‘usual care’ in the NHS.

LSVT is a highly protocolised, intensive intervention, tailored to address individuals’ interests. SNHS SLT is eclectic, tailored to address the presenting speech problems, and has typically been delivered at a low intensity (Miller et al. 2011). Conversations with therapists and a review of the pilot data (Sackley et al. 2018) suggested that these two complex interventions would have distinct and overlapping components; describing them using the TIDieR framework would be essential to understand the nature of the interventions delivered, to assist with transparency about what was (and was not) included in each intervention and its delivery (including home based practice), in order to support interpretation and clinical implementation of the trial results. SLT is subject to NHS service delivery variations and constraints (Miller et al. 2011), so it would also be vital to understand experiences of implementing LSVT at the required intensity.

**FIGURE 2:**
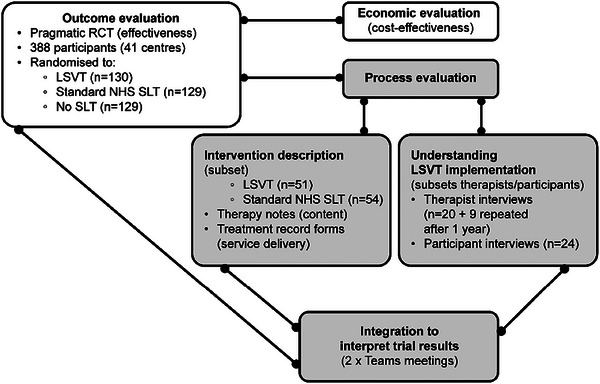
PD COMM trial, including mixed method process evaluation.

PD COMM, therefore, included a two‐part process evaluation, which was conducted independently by separate teams before being integrated. One team described the trial interventions (PE‐ID), while the other included a focus on understanding experiences of LSVT implementation (PE‐IMP) (Figure 2).

### 1.1 Aims

In this paper, we aim to:

1. Describe the content and service delivery components of the PD COMM trial SLT interventions using the TIDieR reporting framework.
2. Identify key similarities and differences in content and service delivery between the LSVT and SNHS SLT interventions.
3. Understand the experiences of implementing LSVT for the trial.
4. Consider how these findings can help to explain the PD COMM trial outcomes.
5. Reflect on the implications of PD COMM for clinical practice and future research.

## 2 Methods

This section covers our pragmatic approach for intervention description (PE‐ID, quantitative) and experiences of LSVT implementation (PE‐IMP, qualitative), which were done in parallel, and how we then integrated these findings to help interpret the main PD COMM trial outcomes. In crafting this paper, we drew on reporting guidelines including TIDieR (Hoffmann et al. 2014) and Good Reporting of a Mixed Methods Study (GRAMMS) (O'cathain et al. 2008).

### 2.1 Intervention Description (PE‐ID)

#### 2.1.1 Data Sources and Transfer

Anonymised Individual Participant Therapy Data were extracted from:

- Therapy notes, which therapists or assistants completed as part of their usual practice after each therapy session: *intervention content data*.
- SLT Treatment Record Forms, which therapists or assistants completed for each therapy session: *service delivery data*.
- Home‐Based Therapy Diaries (home diaries), which were completed by participants +/‐ support from therapists and/or carers: *data on home practice activities*.

NHS staff redacted personal information before copying and sending these data sources to the Birmingham Clinical Trials Unit (BCTU) where it was checked and, if necessary, redacted further before being scanned as a PDF and shared with the PE‐ID team.

A Data Transfer Agreement supported the transfer of the anonymised data to Glasgow Caledonian University (GCU) for data extraction and analysis. The first tranche of data was transferred in November 2019 using a secure, password‐protected data sharing platform. The remainder of the data was transferred in January 2020. All Individual Participant Therapy Data was downloaded on the same day to a secure GCU network drive only accessible to the named members of the research team (A.N., S.D., M.B.).

#### 2.1.2 Sampling

Data availability was mapped (A.N.) to inform systematic and transparent decision making in keeping with the published process evaluation protocol (Masterson‐Algar et al. 2017).

Several factors limited data availability at the time of the PE‐ID. We therefore applied the following data eligibility criteria to support our planned analysis: participants had completed their allocated intervention (LSVT or SNHS SLT) with at least two Treatment Record Forms, associated therapy notes and home diaries available.

#### 2.1.3 Blinding

Details of the SLT intervention content were extracted, so blinding to the participants’ allocation was not possible. As the data was examined without reference to the outcome data, the risk of detection bias was not applicable.

#### 2.1.4 Data Extraction

We used separate spreadsheets to extract data from the therapy notes (intervention content) and the Treatment Record Forms (service delivery) for each SLT intervention.

Each Individual Participant Therapy Data was extracted and profiled on a single row in each spreadsheet. Column headings supported data extraction of therapy activity by categories, thus each row recorded summary data for each participant. Columns provided summary data on therapy activity categories.

To enable comparison between SNHS SLT and LSVT, Individual Participant Therapy Data was entered using the same headings, whether the source was Treatment Record Forms or therapy notes. There were more headings for SNHS SLT home‐based practice as this had the potential to be very varied in comparison to the tight LSVT prescription.

We developed extraction category headings iteratively, then applied them systematically (Appendix 1). In addition to the TIDieR reporting template, these headings were informed by relevant literature, LSVT training and proformas, and the categories in the trial Treatment Record Forms (agreed and piloted previously; Sackley et al. 2018, Appendix 2). They were refined through inductive analysis of eight sets of SNHS SLT therapy notes supplemented by all prescribed content in the SNHS SLT home diaries.

Data extraction was either numerical or categorical. Numerical data were documented for categories such as the number of Treatment Record Forms and the total minutes spent on impairment intervention. Categorical data was used for yes/unreported, or for a list of options, for example, location of intervention (outpatient/home/mixed). Coding instructions supported consistency in data extraction. Where applicable, this included quantification, for example, that ‘mixed’ would apply where either outpatient or home was <80%.

Individual Participant Therapy Data was extracted by team members (A.N., M.B., S.D.). Data extraction from therapy notes, including data relating to prescribed home practice, was completed by an LSVT‐trained speech and language therapist (A.N.) who wrote brief memos to document her observations and reflections. Decision making was informed by regular discussion with another speech and language therapist (M.B.). A coding table was used to describe the scope of each data item category and to record examples.

A second speech and language therapist (M.B.) independently checked 20% of the records. Where necessary, identified discrepancies, errors or omissions were discussed and resolved and data extraction categories refined.

#### 2.1.5 Data Analysis

Simple descriptive statistics were calculated; sum and percentage for categorical data, and sum, mean, median, mode and range (minimum and maximum) for numerical data. We examined this data to identify the key similarities and differences between the content (rationale, procedures, materials, tailoring) and service delivery (provider, delivery, location, regimen) of the two trial interventions. Given the nature of the data and the purpose of the analysis, we had to make a judgement about reasonable cut‐off points. Through discussion, we considered a difference of more than 25 percentage points may represent a key between‐intervention difference, while differences of less than 10 percentage points may represent a key similarity.

### 2.2 Experiences of LSVT Implementation (PE‐IMP)

Investigations of therapist and patient experiences were informed by the Normalisation Process Theory (NPT) (May and Finch 2009), a sociological theory describing the different types of work associated with the adoption of new technology, in this case LSVT. In‐depth qualitative interviews were carried out with a sub‐sample of therapists delivering both interventions when they first joined the trial (*n* = 20) and also after one year (*n* = 9). Interviews with a sub‐sample of patients (*n* = 24) across the three trial arms were also carried out to investigate their perceptions and experiences. All interviews (P.M.A., M.C.) were digitally recorded, fully transcribed, and coded using a theoretical coding framework informed by the NPT constructs; new codes were created for data falling outside of the coding framework to avoid missing important concepts. Findings in relation to experiences of LSVT implementation were summarised for this paper (P.M.A., C.B.). For detailed methods and other information about PE‐IMP, see Sackley et al. (2024a).

### 2.3 Integration and Interpretation (Guest 2013)

The PE‐ID and PE‐IMP analyses were done in parallel before the PD COMM findings were available. The first draft of this paper contained only the PE‐ID methods and results. A.N., M.B., P.M.A., C.B., G.B., and C.H.S. subsequently held two online workshops (over Teams) during which we identified how purposively selected themes from PE‐IMP could increase our understanding of the LSVT implementation. We then explored how the process evaluation findings as a whole might help interpret trial results and inform recommendations for practice and research.

## 3 Findings

In this section, we describe the content and service delivery components of the two interventions in relation to the TIDieR framework (PE‐ID sample), before summarising their key similarities and differences. We then consider our qualitative findings to understand experiences of implementing LSVT from the perspective of therapists and people with Parkinson's (PE‐IMP sample).

### 3.1 Describing Content and Service Delivery of the Trial Interventions (PE‐ID)

#### 3.1.1 Number of Individual Participant Therapy Data

**FIGURE 3:**
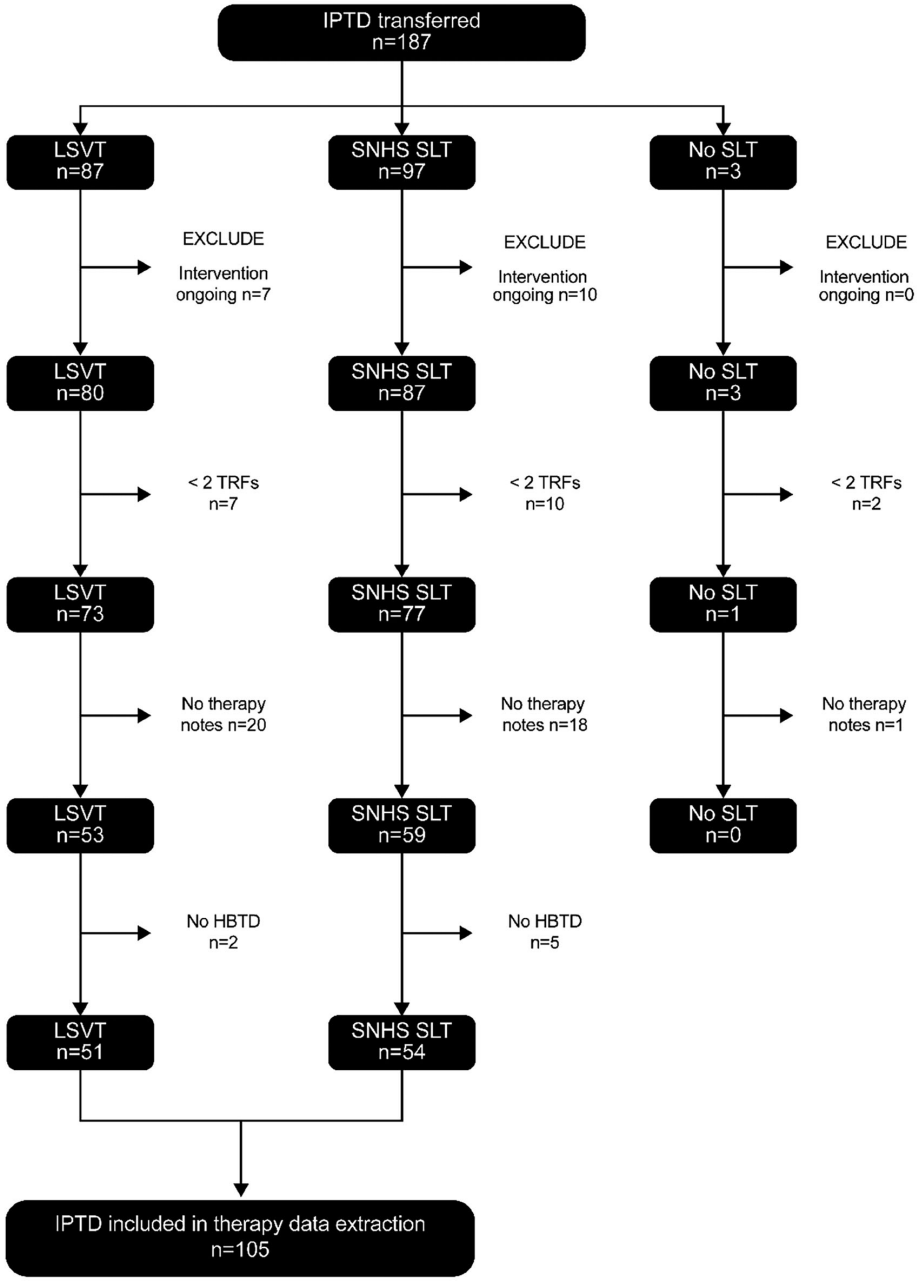
Modified PRISMA 2009 flow diagram. HBTD, Home Based Therapy Diaries; IPTD, Individual Participant Therapy Data; TRFs, Treatment Record Forms.

Of available Individual Participant Therapy Data (*n* = 187), only 105 (LSVT *n* = 51; SNHS SLT *n* = 54) met our inclusion criteria (Figure 3). We carried out a variety of checks using the question ‘what might be missing’ and found no imbalance between Individual Participant Therapy Data for the two interventions.

#### 3.1.2 Content: Why the Therapy Was Being Done

A rationale for most SNHS SLT was recorded from both participants’ and therapists’ perspective (91%, *n* = 49/54 and 87%, *n* = 47/54, respectively). Documentation of a rationale in LSVT therapy notes was less likely (participant perspective 57%, *n* = 29/51; therapist perspective 37%, *n* = 19/51).

Similarly, SNHS SLT intervention goals were commonly recorded (96%, *n* = 52/54), unlike LSVT (41%, *n* = 21/51). Goals from the participant's perspective were recorded across both interventions (SNHS SLT 41%, *n* = 22/54; LSVT 27%, *n* = 14/51) and, where unreported, therapist‐led goals (outcomes, task or session‐based) were recorded more frequently in SNHS SLT (56%, *n* = 30/54) than in LSVT (14%, *n* = 7/51).

Over half the time, both interventions included a documented intervention delivery plan (LSVT 57%, *n* = 29/51; SNHS SLT 56%, *n* = 30/54). In LSVT, the delivery plan was often reported as an explanation to participants of the timetable.

#### 3.1.3 Content: What Materials Were Reported

##### 3.1.3.1 Technologies

Almost all therapy notes (LSVT 98%, *n* = 50/51; SNHS SLT 96%, *n* = 52/54) reported using exercise materials. These included worksheets, lists, pictures for description, reading passages and clients’ own materials such as magazines or books. LSVT‐specific materials included LOUD card prompts and worksheets from the manual. One therapist reported using a ‘tell me about grid’ for the participant to choose conversation topics. Provision of information sheets was rarely documented. However, one SNHS SLT notes listed all information sheets given (with dates), reflecting the local requirement to do so.

Reference to using telephones or video calls as part of intervention (whether in the session or as a home‐based practice task) was made in 69% (*n* = 35/51) of LSVT but only 13% (*n* = 7/54) of SNHS SLT notes. The use of the internet was rarely described. Two examples in SNHS SLT directed participants towards YouTube video demonstrations to learn a technique. There was a difference in reported use of apps between the two interventions (LSVT 37%, *n* = 19/51; SNHS SLT 17%, *n* = 9/54), but the range of apps reported was more varied in SNHS SLT (which uniquely featured Voice Meter Pro, DAF pro, Bla Bla Bla and a trial of ClaroCom as AAC). In contrast, 10 of the 19 reported app uses in LSVT specified Voice Analyst, and one used an app to encourage vocal effort through creating background noise. Parkinson's UK apps and Decibel 10 were reported in both interventions.

No use of AAC materials was reported in LSVT. In SNHS SLT, one trial of an amplifier was reported, but the participant was unable to get funding to purchase it. Use of low technology AAC to support pacing (alphabet chart, pacing board, body parts) was documented in four participants’ notes, and an additional participant reported successfully using Dragon speech‐to‐text software.

One therapist reported exchanging materials with a participant via email, and another loaned a tape recorder and tape to a participant who did not own a smartphone or tablet. Use of laptops, iPads and mobile phones was more commonly reported in LSVT participants’ notes, and there were three examples of the LSVT Homework Helper DVD being loaned, one with a chunky stylus.

##### 3.1.3.2 Biofeedback

It was challenging to extract the use of visual or auditory biofeedback, as it was not always clear in the notes that a tool was being used. Visual biofeedback was described in 86% (*n* = 44/51) LSVT and 57% (*n* = 31/54) SNHS SLT interventions. Auditory biofeedback was reported in 41% (*n* = 21/51) LSVT and 37% (*n* = 20/51) SNHS SLT. Use of both visual and auditory biofeedback was reported in 37% (*n* = 19/51) LSVT and 27% (*n* = 14/54) SNHS SLT interventions.

Description of resistance biofeedback was infrequent across both interventions, but may have differed in its purpose by intervention approach. In LSVT, 6% (*n* = 3/51) used resistance for bearing down or pushing their palms together to achieve maximum phonation. In SNHS SLT, five of the six interventions incorporating resistance (11%) used a tube or straw in water. Reported use of tactile biofeedback was limited to SNHS SLT (15%, *n* = 8/54), where participants were asked to use their hands to feel for vocal vibrations or chest movement.

##### 3.1.3.3 Numerical Measurement

Numerical measurement was common in LSVT, using a tool such as a sound meter (78%, *n* = 40/51) or Companion Software (16%, *n* = 8/51). This compares with 52% (*n* = 28/54) and 4% (*n* = 2/54) of participants receiving SNHS SLT.

Self‐rating scales were reported in 53% (*n* = 27/51) of LSVT and 39% (*n* = 21/54) of SNHS SLT notes. Self‐rating of effort is a required component of LSVT, although one therapist noted that an LSVT participant was unable to use a numerical or Likert scale to rate their effort. Few records documented using observer ratings to support treatment (LSVT 2%, *n* = 1/51; SNHS SLT 4%, *n* = 2/54).

#### 3.1.4 Content: What Intervention Procedures Were Reported

##### 3.1.4.1 Named Therapy Approaches

Other than LSVT, few named therapy approaches were documented. Therapists described using a Care Aims approach (McCarthy et al. 2001) with seven participants (13%) receiving SNHS SLT compared to two (4%) participating in LSVT. Voice techniques referred to in SNHS SLT related to ‘Semi‐Occluded Vocal Tract’ (SOVT) and ‘the Accent method’. In the LSVT records, there was one reference to the ‘giggle posture’ for voice strain, while one SNHS SLT described using ‘glottal strokes’, an exercise to bring the vocal folds together. SNHS SLT descriptions also included a report of ‘bilateral facial massage’, a ‘brushing technique’ for the face, and the use of ‘solution focused’ materials.

##### 3.1.4.2 Intervention Targets (Speech Subsystems)

The LSVT interventions targeted phonation (98%, *n* = 50/51); we considered one ‘unreported’ due to limited therapy documentation. Rarely, therapists also targeted relaxation, posture, rate or other prosody explicitly. Of the 51 LSVT notes, 86% (*n* = 44) had one speech impairment target, and 8% (*n* = 4) had two (with the remainder having none (*n* = 1) or multiple (*n* = 2) targets documented). In contrast, 52% of SNHS SLT interventions focused on three or more speech subsystems (*n* = 28/54), followed by two subsystems (28%, *n* = 15/54). The remaining 19% (*n* = 10/54) focused on one speech subsystem impairment target. The most common speech subsystems focused on within SNHS SLT were phonation (78%, *n* = 42/54) and breath support (67%, *n* = 36/54). Where there was one speech target, these were phonation (*n* = 5/10), breathing (*n* = 4/10), and speech rate (*n* = 1/10).

##### 3.1.4.3 Voice and Speech Cues

Use of ‘Loud’ or ‘Think Loud’ was documented with 98% (*n* = 50/51) of LSVT participants and was typically the only cue recorded (96%, *n* = 49/51).

In comparison, there was more variation in SNHS SLT cues, although 70% (*n* = 38/54) reported ‘Loud’ or ‘Think Loud’ and 22% (*n* = 12/54) reported another cue referencing volume (such as ‘strong’ voice). Other cues documented in SNHS SLT referenced articulation (‘clear’ 26%, *n* = 14/54), exaggerated movements (28%, *n* = 15/54); pace (‘slow’ 17%, *n* = 9/54) or ‘chunking’ of speech into smaller groups of words with pauses (9%, *n* = 5/54). There was one example (2%) of a therapist cueing a ‘forward focus’ for vocal projection.

##### 3.1.4.4 Vocal Activities

We categorised descriptions of vocal activities as: non‐speech oro‐motor exercises (NSOMEs), consonant/vowel/consonant plus vowel combinations (C/V/CV), automatic speech, vocal play, hierarchical manipulation of utterance length and complexity, functional or everyday phrases, and interactions with others. LSVT training suggests that five of these categories should form part of LSVT interventions, while NSOMEs and automatic speech would not. Sixty‐nine per cent (*n* = 35/51) of SNHS SLT mentioned four vocal activity categories. An additional 22% (*n* = 11/51) reported using three categories.

In LSVT, there was one mention of NSOMEs, none of automatic speech, and only 12% (6/51) of vocal play. In contrast, 98% (*n* = 50/51) reported using C/V/CV, 96% (*n* = 49/51) functional phrases and hierarchical speech, and 67% (*n* = 34/51) interaction. SNHS SLT described more use of vocal play (30%, *n* = 16/54) and automatic speech activities (28%, *n* = 15/54), but fewer interactive activities (22%, *n* = 12/54) than LSVT interventions. NSOMEs were reported in 19% (*n* = 10/54) of SNHS SLT, although this appeared to be addressing drooling rather than speech. SNHS SLT interventions described hierarchical activities (83%, *n* = 45/54), C/V/CV (69%, *n* = 37/54), and functional everyday phrases (50%, *n* = 27/54).

##### 3.1.4.5 Practice Structure

Daily and hierarchical practice (both 94%, *n* = 48/51), applied tasks and home‐based practice review (both 86%, *n* = 44/51) were commonly reported in LSVT documentation. Deliberate multi‐tasking (e.g., practising a loud voice while making coffee) was less reported (29%, *n* = 15/51). Although documented home‐based practice review was similar (83%, *n* = 45/54), SNHS SLT percentages were lower across other categories: 85% (*n* = 46/54) daily tasks, 59% (*n* = 32/54) hierarchical tasks, 35% (*n* = 19/54) applied tasks and 13% (*n* = 7/54) multi‐tasking.

##### 3.1.4.6 Other Intervention Targets

Some therapists reported intervening to address Parkinson's‐associated linguistic or cognitive impairment (LSVT 2%, *n* = 1/51; SNHS SLT 13%, *n* = 7/54).

Therapists frequently recorded activities to increase participants’ insight into their Parkinson's‐related communication challenges. Re‐training of sensory perception was reported in 92% (*n* = 47/51) of LSVT (where it is termed ‘calibration’) and 76% (*n* = 41/54) of SNHS SLT notes.

Sixteen per cent (*n* = 8/51) of LSVT and 22% (*n* = 12/54) of SNHS SLT notes described environment modification strategies (e.g., reducing background noise) or deliberately using background noise to encourage participants’ loud voices.

Though examples were rare, we extracted information on techniques addressing the social, psychological, emotional or spiritual needs of people adjusting to, coping with and planning for a progressive condition. Interventions included self‐belief sentence practice exercises, identifying barriers to loud voice use outside therapy and self‐talk strategies to overcome them, and providing national, local and lifestyle management course details. Such psychosocial targets were reported in 10% (*n* = 5/51) LSVT and 26% (*n* = 14/54) SNHS SLT notes.

Promoting self‐management was similarly rarely reported as an *explicit* target. However, drawing on Yorkston et al. (2017a), we sought descriptions of helping participants to develop their own problem‐solving skills; educating or preparing them to make decisions; connecting them to resources; helping them prepare for interactions with other healthcare providers; and supporting them with feasible action plans, such as for maintenance. Examples included asking ‘If you were someone else, what would you say to the current you?’; of three therapy weeks, 2 weeks were directed home practice followed by a self‐directed week; tallying successful phone calls to rebuild confidence; discussing situations where a participant might forget to use their loud voice (e.g., throwaway comments, rummaging in a cupboard); and probing how a participant would generate conversational opportunities over a weekend where they had no activities planned. Such self‐management activities were reported in 41% (*n* = 21/51) LSVT and 54% (*n* = 29/54) of SNHS SLT.

#### 3.1.5 Content: Tailoring

##### 3.1.5.1 Tailoring to the Person

Tailoring materials to a client's interests (including cars, thrillers, DIY, yoga) was described in 75% (*n* = 38/51) of LSVT interventions compared to 35% (*n* = 19/54) of SNHS SLT. Tailoring to a client's interactions (e.g., applied (carryover) tasks such as asking the post office to send a parcel overseas or speaking to an event official) was reported in 67% (*n* = 34/51) LSVT and 37% (*n* = 20/54) SNHS SLT. Descriptions of tailoring to participants’ communication goals, however, (e.g., practising a planned lecture) were reported in 24% (*n* = 12/51) of LSVT interventions and 39% (*n* = 21/54) of SNHS SLT. Although intensive interventions can raise tolerance concerns, the tailoring of intervention to participants’ health was documented in both LSVT (33%, *n* = 17/51) and SNHS SLT notes (39%, *n* = 21/54).

##### 3.1.5.2 Tailoring Using Therapeutic Skills

In LSVT, therapists model communication behaviours (‘do as I do’) and avoid explanation. Modelling was reported in 73% (*n* = 37/51) of LSVT and 54% (*n* = 29/54) of SNHS SLT. Task titration (increasing or decreasing the degree of task difficulty) or facilitating an augmentative task response was described in both (LSVT 96%, *n* = 49/51; SNHS SLT 89%, *n* = 48/54). Advice and educational elements were more commonly reported in SNHS SLT (89%, *n* = 48/54) than LSVT (65%, *n* = 33/51). Across interventions, therapists reported using encouragement (LSVT 45%, *n* = 23/51; SNHS SLT 50%, *n* = 27/54) and described involving others to provide participant feedback (LSVT 31%, *n* = 16/51; SNHS SLT 31%, *n* = 17/54).

#### 3.1.6 Content: Home‐Based Practice

Challenges in documenting adherence to home‐based therapy practice and variation in how the home diaries were completed limited our extraction and interpretation of the information, particularly dosage. We therefore supplemented the home diaries information with therapy notes.

LSVT home‐based practice is highly protocolised for content and dosage and was documented in almost all LSVT notes (98%, *n* = 50/51). Prescriptions reported in the therapy notes included daily ahs, highs, lows, functional (everyday) phrases, hierarchical drills, carryover (applied) tasks and effort rating. The highest report was for prescription of carryover tasks, at 84% (*n* = 43/51) and the lowest for documenting effort rating (24%, *n* = 12/51). Ahs, highs, lows and functional phrases were all reported for 53% (*n* = 27/51).

Home‐based practice prescription was also reported in 98% (*n* = 53/54) of SNHS SLT notes. In SNHS SLT, home diaries were weekly. The average number of home diaries was 5 (median 4.5, range 1–12). The average number of tasks over the course of therapy was 20 (median 15, range 0–70).

We categorised SNHS SLT home‐based practice by the prescription (daily practice), the focus or target (relaxation, posture, breathing, vocal hygiene, LOUD, resonance, clear speech and intelligibility strategies, pacing, prosody, and face/lip/tongue), and methods. Methods included exercises (ahs, everyday (functional) phrases, hierarchical tasks, applied (carryover) tasks, tube resistance), conversation partner tasks, and self‐management activities (preparation, self‐monitoring, education, trialling AAC).

SNHS SLT home‐based practice content was similar to some LSVT components: practice of ahs (44%, *n* = 24/54), everyday (functional) phrases (52%, *n* = 28/54), hierarchical tasks (76%, *n* = 41/54) and applied (carryover) tasks (41%, *n* = 22/54). A LOUD or volume focus was reported in 67% (*n* = 36/54). The prescription structure included 87% (*n* = 47/54) advising daily practice and 91% (*n* = 49/54) prescribing exercises.

Other SNHS SLT home‐based practice was dissimilar to LSVT: a focus on breathing (56%, *n* = 30/54), clear speech (30%, *n* = 16/54), face/lip/tongue (22%, *n* = 12/54), pacing (17%, *n* = 9/54), relaxation (11%, *n* = 6/54), posture or prosody (both 7%, *n* = 4/54) or resonance (4%, *n* = 2/54). Another feature of SNHS SLT home‐based practice was a reported focus on vocal hygiene (11%, *n* = 6/54), although use of water and attention to good quality voice was frequently documented in the LSVT therapy notes.

SNHS SLT home‐based practice prescription reports included a greater range of methods: tube resistance and word generation (each 9%, *n* = 5/54); promoting self‐management (19%, *n* = 10/54) and conversation partner tasks (13%, *n* = 7/54).

#### 3.1.7 Service Delivery: Individual Participant Therapy Data

Individual Participant Therapy Data on service delivery extracted from the Treatment Record Forms is summarised in Table 1. As applicable, we report this for each intervention as Individual Participant Therapy Data and percentage, or as sample total and average.

**TABLE 1:** Service delivery comparison: LSVT and SNHS SLT.

Therapy descriptor	LSVT *n* = 51	SNHS SLT *n* = 54
**Number of therapy sessions (on average) provided** ^a^		
Assessment only session	44 (<1)	63 (1)
Intervention session	763 (15)	260 (5)
**Number of therapy sessions (on average) provided by** ^a^		
Therapist	786 (15)	315 (6)
Therapy assistant	27 (1)	23 (0)
Supervised student	0 (0)	4 (0)
Software based therapy (LSVT Companion)	16 (0)	0 (0)
**Number of SLT professionals involved (%)**		
1 SLT professional	17/51 (33%)	46/54 (85%)
2 SLT professionals	28/51 (55%)	7/54 (13%)
3 SLT professionals	5/51 (10%)	1/54 (2%)
4 SLT professionals	1/51 (2%)	0
**Mode of delivery (%)**		
One‐to‐one	50/51 (98%)	52/54 (96%)
Group therapy	0 (0%)	2/54 (4%)
Mixed one‐to‐one and software (LSVT Companion)^b^	1/54 (2%)	0 (0%)
**Location of intervention delivery (%)**		
Outpatient	38/51 (75%)	40/54 (74%)
Home	5/51 (10%)	6/54 (11%)
Mixed	8/51 (16%)	8/54 (15%)
**Therapy activities in minutes (on average)**	**Minutes**	**Minutes**
Assessment	6,355 (125)	4804 (89)
Goal setting	948 (19)	1425 (26)
Information provision *(participant)*	1,314 (26)	1634 (30)
Information provision *(others)*	253 (5)	279 (5)
Impairment therapy^c^	245 (5)	5890 (109)
Compensatory therapy^c^	32 (1)	1465 (27)
AAC therapy^c^	0 (0)	75 (1)
Generalisation^c^	196 (4)	1442 (27)
Training	22 (0)	61 (1)
LSVT^c^	39,188 (768)	0 (0)
Companion^c^	960 (19)	0 (0)
Indirect and Liaison	832 (16)	305 (6)
Other (*administrative activities*)	10,365 (203)	5477 (101)

^a^ Excluding sessions without direct patient contact, e.g., for phone calls to arrange appointments, drafting reports.

^b^ 8 sessions face‐to‐face plus 8 sessions unsupervised LSVT Companion software.

^c^ Counted as direct intervention activities (although this arguably could have included goal setting and/or information provision, and excluded Companion).

Based on an included sample of 1141 Treatment Record Forms (SNHS SLT *n* = 340; LSVT *n* = 801), we reviewed the therapy intervention details recorded by therapists. On average, 15 intervention sessions were provided for LSVT, compared to 5 for SNHS SLT.

#### 3.1.8 Service Delivery: Who Provided, How, and Where

In our sample, both interventions were predominantly delivered one‐to‐one (LSVT 98%, *n* = 50/51; SNHS SLT 96%, *n* = 52/54) and in outpatient settings (LSVT 75%, *n* = 38/51; SNHS SLT 74%, *n* = 40/54).

Therapists delivered most sessions (LSVT average 15; SNHS SLT average 6) though some were delivered by assistants. Computer software was rarely reported, and only in the context of LSVT. Most SNHS SLT was provided by a single therapist (85%, *n* = 46/54 vs. LSVT 33%, *n* = 17/51). In contrast, LSVT interventions were often delivered by two or more therapists (67%, *n* = 34/51 vs. SNHS SLT 15%, *n* = 8/54).

#### 3.1.9 Service Delivery: When and How Much of What Was Provided (Therapy Dosage and Activities)

On average, in LSVT more minutes reportedly focused on assessment activities (125 min) versus SNHS SLT (89 min). In SNHS SLT, there was a higher average time on goal‐setting activities (26 min) versus LSVT (19 min). Both interventions had a similar average time on information provision. On average, LSVT Treatment Record Forms reported most therapy time spent specifically on LSVT activities (787 min = 13 h 7 min), while SNHS SLT was predominantly on impairment (109 min), compensatory (27 min) and generalisation (27 min) therapy activities.

In our sample, the difference in total reported direct intervention dosage (as defined in Table 1, see c) was striking (on average, LSVT 797 min = 13 h 17 min; SNHS SLT 164 min = 2 h 44 min). Assessment and administration are essential underpinning activities for intervention, and again, there were differences. The three main activity times were, in order, the same for both interventions, but average time on each was greater for LSVT. Averages for SNHS SLT Treatment Record Forms were impairment therapy (109 min), administration (101 min) and assessment (89 min), compared to LSVT Treatment Record Forms, which averaged LSVT (768 min), administration (203 min) and assessment (125 min).

#### 3.1.10 Quality Check

Few differences were identified in the data extraction check of 20% of the data. Discrepancies identified with the Treatment Record Forms data (20/616 data items; 3.24%) were distributed across both interventions (6 in SNHS SLT; 14 in LSVT). These discrepancies were checked with few (4 SNHS SLT; 11 LSVT) errors in the original data extraction (2.43%). Data extraction from the therapy notes was more challenging with data recorded in free text, on occasion handwritten notes, and data items that drew on data from across the Treatment Record Forms, therapy notes and home diaries. On checking the data extraction across 1386 data items, we identified 5.99% omitted data items from the first round of data extraction. Errors or omissions identified in the data check were corrected in the main dataset prior to reporting here.

### 3.2 Similarities and Differences: What Was LSVT and What Was SNHS SLT (PE‐ID)?

The key difference between the two SLT interventions was ‘dosage’, with an average 797 min = 13 h 17 min LSVT compared with 164 = 2 h 44 min SNHS SLT reported. The rationale for SNHS SLT dosage was unclear from therapy notes (e.g., ‘Explained need to develop volume and prosodic skills and enhance word recall. Patient to attend 4 therapy sessions at X Hospital on a fortnightly basis’).

To explore other similarities and differences between the interventions, we considered content (materials, procedures, tailoring) and service delivery (who, how and where) components.

Key similarities included the level of use of speech/voice exercise resources and auditory biofeedback, reviewing home‐based practice, and environmental modification strategies. With tailoring, both interventions reported varying the level of challenge by participant response, using encouragement, tailoring therapy to the participant's health, and involving others to provide feedback.

**FIGURE 4:**
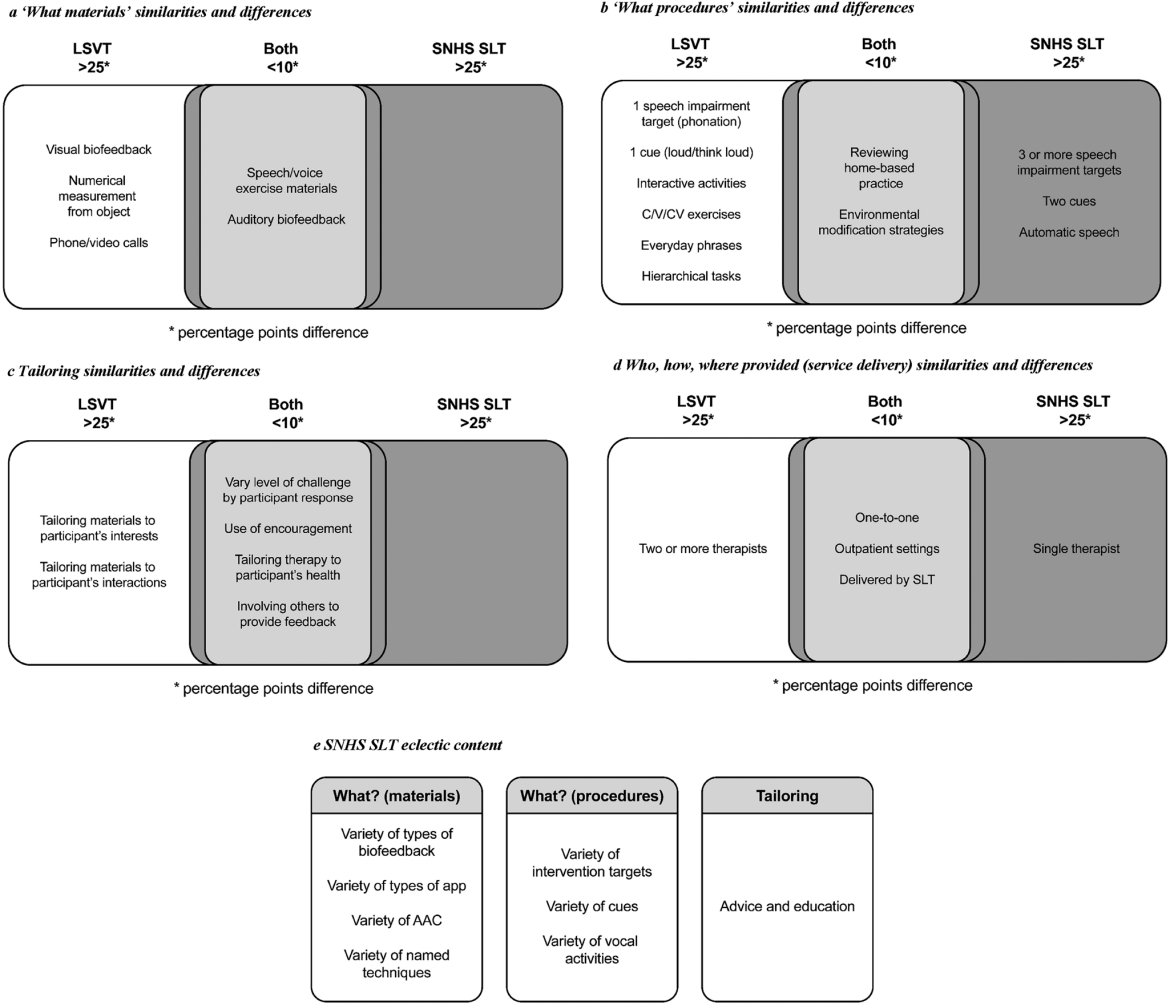
(a) ‘What materials’ similarities and differences. (b) ‘What procedures’ similarities and differences. (c) Tailoring similarities and differences. (d) Who, how, and where provided (service delivery) similarities and differences. (e) SNHS SLT eclectic content.

In terms of materials (Figure 4a), LSVT was distinguished by its use of visual biofeedback, numerical measurement tools, and phone/video calls. No SNHS SLT materials component met our >25 percentage points of difference threshold.

For procedures (Figure 4b), LSVT had a single impairment target (phonation) with a single cue (‘think loud’) and used interactive activities, C/V/CV exercises, everyday phrases and hierarchical tasks. In comparison, SNHS SLT featured three or more speech impairment targets and two cues, and used automatic speech.

With tailoring (Figure 4c), LSVT tailored materials to a participants’ interests and interactions. No SNHS SLT tailoring components met our >25 percentage point difference threshold.

Service delivery (who, how and where provided) (Figure 4d) was similar; mostly one‐to‐one in outpatient settings with an SLT. LSVT was distinguished by two (or up to four) therapists for delivery. SNHS SLT was typically delivered by a single therapist.

In summary, while LSVT as a package was only in the LSVT arm, both PD COMM SLT interventions explored in the included Individual Participant Therapy Data focused on impairment therapy. For a pragmatic trial, LSVT's prescribed content and intensive service delivery in this sample appeared to be in line with the developers’ requirements (TIDieR category ‘How well’). In contrast, SNHS SLT was eclectic (Figure 4e) and characterised by low dosage.

### 3.3 Experiences of LSVT Implementation (PE‐IMP)

In qualitative interviews, therapists and participants had the opportunity to talk about their experiences of the trial and its interventions. This included the kinds of ‘work’ they—and others—had to do to deliver LSVT as intended, as well as how this process made them feel. For therapists, it was also an opportunity to reflect on similarities and differences between intervention (both LSVT and SNHS SLT) as delivered during versus prior to the trial.

#### 3.3.1 Therapist Perspectives

The therapists we spoke to had a pragmatic approach to ‘*putting together’* the content for SNHS SLT and broadly felt confident that both interventions were ‘*different enough’*. Effective mechanisms of LSVT identified by therapists concentrated on treatment intensity, repetition, and prescription. Other effective mechanisms reported by therapists included social aspects of LSVT, such as ‘*attending therapy*’ and ‘*meeting other people*’ on an intensive basis. This generally differentiated LSVT from the more ‘*flexible*’ SNHS SLT.

The LSVT brand was positively regarded; training was seen as ‘*intensive*’, ‘*motivational*’ and ‘*inspiring*’, and a positive addition to a therapist's portfolio. Training increased knowledge and practical skills and drove implementation by helping therapists to differentiate LSVT from other practices, and to ‘*rejig the teams*’ to increase patients’ access to LSVT. In addition to trial staff, therapy leads played a role in implementation through developing flowcharts and guides to direct SLT practice. Having another therapist to ‘*pair up with*’ was seen as helpful in delivering LSVT. Whereas some therapists had prior experience of LSVT, pre‐trial delivery often comprised LSVT principles rather than LSVT; it was less intensive, more flexible, embedded within other interventions, and described, for example, as ‘*loud therapy’* that was delivered once or twice a week.

Available clinic space, together with increases in staffing and uptake of LSVT training, were both drivers of the roll‐out of LSVT within the context of the trial and linked to ongoing implementation. Some therapists felt that elements of LSVT could be delivered digitally to increase patients’ access and ease of engagement. Most therapists referred to the potential benefits of engaging therapy assistant staff in the delivery of aspects of LSVT, with any risks to treatment fidelity mitigated by its structured nature.

Many therapists referred to patients who benefited from LSVT as ‘*motivated*’ and ‘*active*’. However, the trial was felt to change who would usually be offered LSVT, in particular patients for whom therapists would have previously had concerns about ‘*stamina*’ and ‘*motivation*’. Patients were felt to require a sufficient literacy and ability to retain information, and therapists found that dealing with patients’ ‘*cognitive impairments was built into*’ LSVT.

#### 3.3.2 Participant Perspectives

Most participants we spoke to had wanted to be randomised to LSVT. Those who had hoped they would not be randomised to LSVT had worried about the level of commitment and whether their symptoms were sufficient to merit it. LSVT was described as ‘*different*’ and ‘*American*’. Although this appeared to generate interest and enthusiasm amongst trial participants, other words used to describe LSVT were ‘*intense*’, ‘*concentrated*’ and ‘*tiring*’, and for those who found benefit, ‘*a useful technique*’ and ‘*not being a big ask*’.

‘*Moderating and tailoring*’ activities was a feature of life with Parkinson's, in addition to dealing with symptoms. The additional work associated with engaging with LSVT included ‘*making time in the day*’ for exercises described as focusing on voice strength and quality, in addition to other rehabilitation activities that had been advised for other Parkinson's‐related problems. The systematic and structured design and ‘*discipline*’ of LSVT appealed; some described practice as ‘*proper homework*’. Practice was also described as ‘*perplexing*’ and ‘*frustrating*’; practical challenges were accessing SLT appointments, which the support of family members appeared to mitigate, and the associated travel costs.

Participants felt supported by SLTs (e.g., by providing help with home‐based diaries and trial paperwork), and this increased their level of motivation and engagement in the trial. Driving engagement in LSVT was also linked to a sense of not losing perceived functional gains in speech, ‘*keeping a record*’ of practice, or not wanting to ‘*let down*’ the therapist in maintaining practice exercises. Significant events, for example, a death in the family, could impact negatively on practice maintenance. Support from family and friends (but mainly spouses), together with peer and patient support groups, was highlighted as helpful in developing insight and understanding of LSVT.

Effective mechanisms of LSVT included better understanding of the ‘*mechanics of producing sound*’, and speech preparation, for example, before a social event or personally challenging situation (e.g., ‘*in cold weather*’), and being more conscious about speaking, for example, in terms of real‐time consideration of volume and pacing. Participants who had more severe symptoms prior to taking part in the trial reported an impact of LSVT versus participants with mild voice symptoms reported less/no impact. Benefits described included a sense of ‘*confidence*’ with speech, improvements in voice strength, and ‘*being heard’* in family and other social situations.

## 4 Discussion

The PD COMM process evaluation investigated a sub‐sample of routine and trial‐specific data to describe and compare the trial interventions (LSVT and SNHS SLT) using simple descriptive statistics (PE‐ID). A separate team drew on relevant theory (May and Finch 2009) and conducted qualitative interviews with therapists and participants to understand experiences of implementation, with the focus on the experimental intervention, LSVT (PE‐IMP). Although there were similarities between the two active interventions, our analysis highlighted differences that inform clinical insights into the trial outcomes (French et al. 2020).

A key PD COMM trial finding was*, 3 months after randomisation, LSVT was more effective than no SLT (control) and SNHS SLT at reducing participant‐reported impact of Parkinson's‐related dysarthria, and this benefit persisted at least 12 months after starting treatment* (Sackley et al. 2024b). LSVT has a substantial, incrementally developed body of effectiveness literature but has not been tested previously in a large, robust pragmatic trial using a range of patient‐reported outcome measures. Importantly, our analysis suggests we can be reasonably confident that LSVT content and intensity were largely delivered per protocol, while effective mechanisms of LSVT identified by both therapists and participants were its highly structured design, repetitive nature, practise requirements, and its focus on volume.

The difference in total reported direct intervention dosage (as defined in Table 1) may in itself provide sufficient explanation. However, comparing LSVT to SNHS SLT content highlighted other possible clues about LSVT's effectiveness. In SNHS SLT, therapists targeted phonation, used ‘loud’ cueing, described vocal activities (hierarchical, C/V/CV, functional everyday phrases) and re‐trained sensory perception. However, compared to LSVT, SNHS SLT included far less use of telephones or video calls (materials), of interactive activities (procedures), and of tailoring materials to a client's interests and social life. These latter components—practised at a relevant intensity—may contribute to more effective carry‐over and maintenance of skills by bridging the gap between drills and real‐life interactions, as well as fostering therapeutic relationships.

Therapists’ reports of how they incorporated LSVT principles pre‐trial—‘loud therapy’ embedded within other interventions and delivered once or twice a week—are in line with previously reported routine practice in United Kingdom (Miller et al. 2011) and Australian (Swales et al. 2019) surveys. Intensity of SLT interventions is increasingly recognised as a vital component of intervention effectiveness (Brady et al. 2022, Sugden et al. 2018). However, in Swales et al. (2019), 90% of LSVT LOUD‐certified therapists reported delivery issues due to service factors, including allocated time, caseload sizes, and the number of therapists working part‐time. Without the support of their service, NHS speech and language therapists have limited agency to vary logistical components of intervention (Nicoll et al. 2021), but PD COMM provided participating services with an expectation that they would deliver LSVT at the required intensity. To achieve this, 67% of LSVT interventions were reported as involving two or more therapy staff compared to only 15% in the SNHS SLT arm. Using this arrangement to increase intervention intensity is, however, unlikely to be effective on its own; the collective endeavour was facilitated by the positive regard for mandatory LSVT training, helpful practice documentation, and the structured, prescribed nature of the intervention. Indeed, in content, service delivery and experience of implementation, we were struck by the profoundly social nature of the LSVT intervention and brand.

Overall, our results show that LSVT is a homogenous multi‐component intervention, with a number of factors around its implementation that may influence motivation, confidence, and investment in the outcome. It has been recognised as ‘a selective and elective therapy’ in routine practice (Wight and Miller 2015), and we were aware anecdotally from site reports that the required intensity influenced some patients’ decisions about participating in the trial. It was clear from therapy notes and interviews that some therapists felt patients categorised as active or motivated were most likely to benefit from LSVT. However, the structured implementation of LSVT within the trial participant eligibility criteria meant that it was offered widely, including to patients who some therapists would ordinarily have felt were not good candidates for LSVT assessment. Our findings suggest that, to address such barriers to access, any roll‐out of LSVT should take an inclusive approach but, as it can feel ‘different’ and ‘American’, NHS therapists may need to explicitly address the cultural experience of LSVT with some clients (Nicoll et al. 2021) in addition to perception of their own vocal loudness level.

Although the PD COMM pilot demonstrated that both interventions may be effective in an adequately powered trial (Sackley et al. 2018), there was *a lack of evidence for SNHS SLT effectiveness at group level as provided in the trial*. Our process evaluation offers some explanation. SNHS SLT reflected a broad range of clinical approaches, treatment interventions and materials at a low intensity.

The rationale for SNHS SLT intensity was unclear from therapy notes and may have reflected local practice rather than individual need. We were conscious that therapy notes contain what a therapist is required to document and what they choose to document. We also noticed variation between therapists and types of notes (e.g., structured electronic to freehand). Boa et al. (2018) understood variation in goal setting practice through comparing interviews, observations and case note data for each practitioner, and we would consider this type of triangulation for future process evaluations.

Like LSVT, SNHS SLT focused on exercises to address dysarthric impairment. It is possible that this was not just a reflection of routine practice, but partly an artefact of the trial design. The Voice Handicap Index as the primary outcome measure may have privileged direct intervention for dysarthric impairment rather than, for example, addressing cognitive‐linguistic targets or interaction. We had also anticipated more group intervention being reported in SNHS SLT, but the logistics (including staffing, accommodation and sufficient candidates) may have reduced opportunities to coordinate and offer it within the timelines of the trial.

SNHS SLT notes were, however, more likely than LSVT to document cognitive‐linguistic and psychosocial targets (although numbers were small) and promotion of self‐management. Uniquely, they also reported low technology AAC and specific voice therapy techniques to address the variety of clients’ communication needs. We hope a future pragmatic trial can be designed to compare manualised interventions which have a different theoretical basis and structure (Sonneville‐Koedoot et al. 2015).

Having a range of effective interventions beyond LSVT is especially critical as there are financial and social costs of therapy engagement for patients and their families. PD COMM found that, *3 months after randomisation, carer‐reported quality of life was similar for those in the LSVT and no SLT (control) arms, and better than for those in the SNHS SLT group*. While we have no clear explanations for this finding, memos (A.N.) suggest that beneficial outcomes may mitigate the burden on spouses (e.g., an LSVT therapy note recorded feedback from a spouse that they were laughing more, she hears him singing about the house like he used to do, and she doesn't talk for him now), that the regularity of LSVT contact may enable therapists to spot escalation of non‐motor/health issues and make timely onward referrals, and the positive simplicity of ‘Think LOUD’ (rather than the more negative ‘don't mumble’) may help carers feel less frustrated. Baylor et al. (2024) identified that, to reduce the burden on spouses/partners, the type of SLT support needs to change depending on symptoms and progression, especially beyond the early stages. While not part of the NHS practice identified in this trial, research into newer approaches such as family‐ and interaction‐centred interventions (e.g., Clay et al. 2024) may help to address this need.

*Neither LSVT nor SNHS SLT was cost‐effective as delivered in PD COMM*. This was explored in the trial‐based economic evaluation (Sackley et al. 2024a), but we note that the average time spent on administrative activities was greater in LSVT (203 min) than SNHS SLT (101 min). Some of this work (e.g., around appointments) could be suitable for administrative support. Therapists highlighted the potential benefits of both increasing the skill‐mix of LSVT delivery through the deployment of therapy assistants and the use of digital technology as ways of increasing engagement whilst maintaining treatment fidelity and keeping costs down.

A scoping review suggested that interest in technology for service delivery is relatively greater in Parkinson's than in other clinical populations (Theodoros et al. 2019). With that in mind, it is perhaps surprising that reported use of technology was not higher. However, even in a self‐selecting sample of experienced therapists in the United States and Canada (*n* = 111), fewer than half had ever prescribed an amplification device compared to over three‐quarters who reported using a behavioural intervention such as LSVT (Gates et al. 2024). Barriers included the large number of devices with a range of features in an unregulated market, costs, and uncertainties over client preferences. Use of technology was also infrequently reported in a recent US study of home practice programme provision for people with aphasia (*n* = 80) (Brown et al. 2021). Remote tele‐therapy has increased since the start of the Covid‐19 pandemic (Chadd et al. 2021), and developments in artificial intelligence (AI) should make it easier and quicker to prepare materials and procedures to suit a client's interests and interactions. However, a long history of implementation science literature cautions against underestimating the costs and barriers to use of digital technology in routine practice (Greenhalgh et al. 2017).

## 5 Strengths and Limitations

Our mixed methods process evaluation used the TIDieR framework to describe and compare the PD COMM trial interventions, complemented by a theoretically‐informed analysis of the additional work of implementing LSVT. It added value to this large pragmatic RCT by offering reasons for the trial results and an understanding of their applicability, as well as informing post‐trial steps for research and practice (French et al. 2020).

Using the same Individual Participant Therapy Data categories for intervention description of both interventions addressed a criticism of pragmatic RCTs (Nicholls et al. 2020). While surveys of reported practice are useful, routine contemporaneous therapy notes proved a rich and novel source of what was actually delivered to an individual participant from a therapist's perspective.

Although it was a pragmatic trial, a request not to use LSVT within SNHS SLT may have underplayed the extent to which, in practice, therapists use techniques and activities that are in common with the LSVT approach; similarly, a request to use LSVT as prescribed may have overplayed the extent to which it is used as intended in practice. However, both the branding of LSVT, and its difference from the less intensive and more flexible SNHS SLT, appeared to be key in enabling therapists to differentiate LSVT from other approaches.

Incompatible timelines and bureaucratic barriers limited the process evaluation teams’ access to participants, therapists and data. As a consequence, our samples were smaller than anticipated, although they provide a fair picture of trial interventions as provided and experienced before the start of the Covid‐19 pandemic. Challenges in documenting adherence to home‐based therapy practice may have been avoided by more comprehensive piloting of the paperwork.

In this paper, we focused only on the PE‐IMP findings related to LSVT implementation. Reflecting trial design over 10 years ago, PE‐IMP focused on explicating the ‘experimental’ intervention (LSVT). At that time, it was not considered necessary to do the same with ‘control’ interventions (including ‘usual care’ such as SNHS SLT). The trial has given us a greater understanding that LSVT, SNHS SLT or no dysarthria intervention may all constitute ‘usual care’ in the NHS, depending on local practice. Given improved insights over recent years into the importance of design choices around control interventions, future trials should aim to close this gap in understanding.

## 6 Conclusions

In this sample, LSVT was largely delivered per protocol. High intensity was a key characteristic, commonly achieved by two or more therapists delivering LSVT. Any roll‐out of LSVT needs service support and coordination, and should take an inclusive approach to avoid exacerbating health inequalities. SNHS SLT was eclectic, reflecting a broad range of clinical approaches at a low intensity. Future research could explore a rationale for dosage, more explicit tailoring to individuals and their families, and implementation of interventions that have a different focus.

The findings of our process evaluation contribute to understanding how services can implement LSVT and other intensive interventions as part of routine NHS service delivery. They also start a critical conversation about how we deliver the benefits of LSVT in a cost‐effective manner and build from SNHS SLT practice to develop a range of evidence‐based, implementable alternatives that will benefit people with Parkinson's and their families as their communication support needs change across the course of their disease.

## Ethics Statement

The trial was approved by the West Midlands—Coventry and Warwickshire Research Ethics Committee (15/WM/0443), and participating NHS Trust Research and Development Departments.

## Conflicts of Interest

Avril Nicoll and Gillian Beaton received training in LSVT LOUD from LSVT Global. Marian Brady, Christina H Smith and Gillian Beaton are speech and language therapists, and Avril Nicoll was a speech and language therapist until 31 July 2023.

## Supporting information




**Supplementary Appendix 1**: PE‐ID coding guide (TIDieR: what materials, what procedures, tailoring)
**Supplementary Appendix 2**: Broad categories of SLT therapy sessions documented in TRF

## Data Availability

All requests for access to PD COMM data should be submitted to Chief Investigator Catherine M Sackley. Access to anonymised patient‐level data with a data dictionary may be granted following review, no earlier than six months after this publication, with no end date. Proposals for data access will need to describe how the data will be used. Transfer of data will be by a secure method and only after approval by the Trial investigator team.

## References

[jlcd70084-bib-0001] Baylor, C. , K. J. Cook , and M. J. Mcauliffe . 2024. “Take Us Into Account: Perspectives of Family Members of People With Parkinson's Disease Regarding Speech‐Language Pathology Intervention.” American Journal of Speech‐Language Pathology 33: 736–755. 38092050 10.1044/2023_AJSLP-23-00273

[jlcd70084-bib-0002] Boa, S. , E. Duncan , E. Haraldsdottir , and S. Wyke . 2018. “Patient‐Centred Goal Setting in a Hospice: A Comparative Case Study of How Health Practitioners Understand and Use Goal Setting in Practice.” International Journal of Palliative Nursing 24: 115–122. 29608386 10.12968/ijpn.2018.24.3.115

[jlcd70084-bib-0003] Brady, M. C. , M. Ali , K. Vandenberg , et al. 2022. “Dosage, Intensity, and Frequency of Language Therapy for Aphasia: A Systematic Review–Based, Individual Participant Data Network Meta‐Analysis.” Stroke; A Journal of Cerebral Circulation 53: 956–967. 10.1161/STROKEAHA.121.035216 PMC8884127 34847708

[jlcd70084-bib-0004] Brown, E. V. D. , S. E. Wallace , and Q. Liu . 2021. “Speech‐Language Pathologists' Practice Patterns When Designing Home Practice Programs for Persons With Aphasia: A Survey.” American Journal of Speech‐Language Pathology 30: 2605–2615. 34694899 10.1044/2021_AJSLP-20-00372

[jlcd70084-bib-0005] Carding, P. , and R. Hillman . 2001. “More Randomised Controlled Studies in Speech and Language Therapy.” BMJ: British Medical Journal 323: 645–646. 11566815 10.1136/bmj.323.7314.645 PMC1121222

[jlcd70084-bib-0006] Chadd, K. , K. Moyse , and P. Enderby . 2021. “Impact of COVID‐19 on the Speech and Language Therapy Profession and Their Patients.” Frontiers in Neurology 12: 629190. 33679590 10.3389/fneur.2021.629190 PMC7930219

[jlcd70084-bib-0007] Clay, P. , T. Walton , E. Malin , M. Hutchinson , K. Levitt , C. Williams , H. Crouch , S. Beeke , and S. Bloch . 2024. “Better Conversations With Parkinson's: Co‐Production of a Novel Speech and Language Therapy Intervention With People Living With Parkinson's.” Research for All 8, no. 1: 10.14324/RFA.08.1.07.

[jlcd70084-bib-0008] French, C. , H. Pinnock , G. Forbes , I. Skene , and S. J. C. Taylor . 2020. “Process Evaluation Within Pragmatic Randomised Controlled Trials: What Is It, Why Is It Done, and Can We Find It?—A Systematic Review.” Trials 21: 916. 33168067 10.1186/s13063-020-04762-9 PMC7650157

[jlcd70084-bib-0009] Gates, K. , T. Knowles , H. Mach , and J. Higginbotham . 2024. “Clinical Insights Into the Use of Speech Amplification Devices for Managing Hypophonia: Interviews With Speech‐Language Pathologists.” American Journal of Speech‐Language Pathology 33: 1639–1661. 38512013 10.1044/2024_AJSLP-23-00396

[jlcd70084-bib-0010] Gillivan‐Murphy, P. , N. Miller , and P. Carding . 2019. “Voice Treatment in Parkinson's Disease: Patient Perspectives.” Research and Reviews in Parkinsonism 9: 29–42.

[jlcd70084-bib-0011] Glover, L. , C. Dixon , C. Kobylecki , and F. J. R. Eccles 2023. “Parkinson's and The Couple Relationship: A Qualitative Meta‐Synthesis.” Aging & Mental Health 27: 2420–2429. 37354064 10.1080/13607863.2023.2227119

[jlcd70084-bib-0012] Greenhalgh, T. , J. Wherton , C. Papoutsi , et al. 2017. “Beyond Adoption: A New Framework for Theorizing and Evaluating Nonadoption, Abandonment, and Challenges to the Scale‐Up, Spread, and Sustainability of Health and Care Technologies.” Journal of Medical Internet Research [Electronic Resource] 19: e367. 29092808 10.2196/jmir.8775 PMC5688245

[jlcd70084-bib-0013] Guest, G. 2013. “Describing Mixed Methods Research: An Alternative to Typologies.” Journal of Mixed Methods Research 7: 141–151.

[jlcd70084-bib-0014] Hoffmann, T. C. , P. P. Glasziou , I. Boutron , et al. 2014. “Better Reporting of Interventions: Template for Intervention Description and Replication (TIDieR) Checklist and Guide.” BMJ: British Medical Journal 348: g1687. 24609605 10.1136/bmj.g1687

[jlcd70084-bib-0015] Johansson, I.‐L. , C. Samuelsson , and N. Müller 2020. “Patients' and Communication Partners' Experiences of Communicative Changes in Parkinson's Disease.” Disability and Rehabilitation 42: 1835–1843. 30669899 10.1080/09638288.2018.1539875

[jlcd70084-bib-0016] Levy, E. S. , G. Moya‐Galé , Y. H. M. Chang , et al. 2020. “The Effects of Intensive Speech Treatment on Intelligibility in Parkinson's Disease: A Randomised Controlled Trial.” Eclinicalmedicine 24: 100429. 32639484 10.1016/j.eclinm.2020.100429 PMC7327886

[jlcd70084-bib-0017] Loudon, K. , S. Treweek , F. Sullivan , P. Donnan , K. E. Thorpe , and M. Zwarenstein 2015. “The PRECIS‐2 Tool: Designing Trials That Are Fit for Purpose.” BMJ: British Medical Journal 350: h2147. 25956159 10.1136/bmj.h2147

[jlcd70084-bib-0018] Ludemann, A. , E. Power , and T. C. Hoffmann 2017. “Investigating the Adequacy of Intervention Descriptions in Recent Speech‐Language Pathology Literature: Is Evidence From Randomized Trials Useable?” American Journal of Speech‐Language Pathology 26: 443–455. 28475801 10.1044/2016_AJSLP-16-0035

[jlcd70084-bib-0019] Masterson‐Algar, P. , C. R. Burton , M. C. Brady , et al. 2017. “The PD COMM Trial: A Protocol for the Process Evaluation of a Randomised Trial Assessing the Effectiveness of Two Types of SLT for People With Parkinson's Disease.” Trials 18: 397. 28851443 10.1186/s13063-017-2130-1 PMC5576370

[jlcd70084-bib-0020] May, C. , and T. Finch . 2009. “Implementing, Embedding, and Integrating Practices: An Outline of Normalization Process Theory.” Sociology 43: 535–554.

[jlcd70084-bib-0021] Mccarthy, C. , R. Lacey , and K. Malcomess . 2001. “An Audit of the Application of Care Aims Across the South West Thames Region.” International Journal of Language & Communication Disorders 36: 505–510. 11340840 10.3109/13682820109177937

[jlcd70084-bib-0022] Miller, N. 2017. “Communication Changes in Parkinson's Disease.” Practical Neurology 17: 266–274. 28687681 10.1136/practneurol-2017-001635

[jlcd70084-bib-0023] Miller, N. , K. H. O. Deane , D. Jones , E. Noble , and C. Gibb . 2011. “National Survey of Speech and Language Therapy Provision for People With Parkinson's Disease in the United Kingdom: Therapists' Practices.” International Journal of Language & Communication Disorders 46: 189–201. 21401817 10.3109/13682822.2010.484849

[jlcd70084-bib-0024] Miller, N. , E. Noble , D. Jones , and D. Burn . 2006. “Life With Communication Changes in Parkinson's Disease.” Age and Ageing 35: 235–239. 16540492 10.1093/ageing/afj053

[jlcd70084-bib-0025] Moore, G. F. , S. Audrey , M. Barker , et al. 2015. “Process Evaluation of Complex Interventions: Medical Research Council Guidance.” BMJ: British Medical Journal 350: h1258. 25791983 10.1136/bmj.h1258 PMC4366184

[jlcd70084-bib-0026] Nicholls, S. G. , M. Zwarenstein , and M. Taljaard . 2020. “The Importance of Describing as Well as Defining Usual Care.” The American Journal of Bioethics 20: 56–58. 10.1080/15265161.2019.1687781 31910140

[jlcd70084-bib-0027] Nicoll, A. , M. Maxwell , and B. Williams . 2021. “Achieving ‘Coherence’ in Routine Practice: A Qualitative Case‐Based Study to Describe Speech and Language Therapy Interventions With Implementation in Mind.” Implementation Science Communications 2: 56. 34039444 10.1186/s43058-021-00159-0 PMC8157687

[jlcd70084-bib-0028] O'cathain, A. , E. Murphy , and J. Nicholl . 2008. “The Quality of Mixed Methods Studies in Health Services Research.” Journal of Health Services Research & Policy 13: 92–98. 10.1258/jhsrp.2007.007074 18416914

[jlcd70084-bib-0029] Sackley, C. M. , C. Rick , M. C. Brady , et al. 2024a. “The Effect of Two Speech and Language Approaches on Speech Problems in People With Parkinson's Disease: The PD COMM RCT.” Health Technology Assessment 28: 58. 10.3310/ADWP8001 PMC11474952 39364774

[jlcd70084-bib-0030] Sackley, C. M. , C. Rick , M. C. Brady , et al. 2024b. “Lee Silverman Voice Treatment Versus NHS Speech and Language Therapy Versus Control for Dysarthria in People With Parkinson's Disease (PD COMM): Pragmatic, UK Based, Multicentre, Three Arm, Parallel Group, Unblinded, Randomised Controlled Trial.” BMJ: British Medical Journal 386: e078341. 38986549 10.1136/bmj-2023-078341 PMC11232530

[jlcd70084-bib-0031] Sackley, C. M. , C. H. Smith , C. E. Rick , et al. 2018. “Lee Silverman Voice Treatment Versus Standard Speech and Language Therapy Versus Control in Parkinson's Disease: A Pilot Randomised Controlled Trial (PD COMM pilot).” Pilot and Feasibility Studies 4: 30. 29344405 10.1186/s40814-017-0222-z PMC5763537

[jlcd70084-bib-0032] Schalling, E. , K. Johansson , and L. Hartelius . 2018. “Speech and Communication Changes Reported by People With Parkinson's Disease.” Folia Phoniatrica Et Logopaedica 69: 131–141. 10.1159/000479927 29346787

[jlcd70084-bib-0033] Sonneville‐Koedoot, C. D. , S. A. Adams , E. A. Stolk , and M.‐C. Franken . 2015. “Perspectives of Clinicians Involved in the Restart‐Study: Outcomes of a Focus Group.” American Journal of Speech‐Language Pathology 24: 708–716. 26363127 10.1044/2015_AJSLP-14-0215

[jlcd70084-bib-0034] Sugden, E. , E. Baker , N. Munro , A. L. Williams , and C. M. Trivette . 2018. “Service Delivery and Intervention Intensity for Phonology‐Based Speech Sound Disorders.” International Journal of Language & Communication Disorders 53: 718–734. 29900638 10.1111/1460-6984.12399

[jlcd70084-bib-0035] Swales, M. , D. Theodoros , A. J. Hill , and T. Russell . 2019. “Communication Service Provision and Access for People With Parkinson's Disease in Australia: A National Survey of Speech‐Language Pathologists.” International Journal of Speech‐Language Pathology 21: 572–583. 30496696 10.1080/17549507.2018.1537372

[jlcd70084-bib-0036] Theodoros, D. , D. Aldridge , A. J. Hill , and T. Russell . 2019. “Technology‐Enabled Management of Communication and Swallowing Disorders in Parkinson's Disease: a Systematic Scoping Review.” International Journal of Language & Communication Disorders 54: 170–188. 29923267 10.1111/1460-6984.12400

[jlcd70084-bib-0037] Wight, S. , and N. Miller 2015. “Lee Silverman Voice Treatment for People With Parkinson's: Audit of Outcomes in a Routine Clinic.” International Journal of Language & Communication Disorders 50: 215–225. 25469736 10.1111/1460-6984.12132

[jlcd70084-bib-0038] Wylie, K. , H. M. Carrier , A. M. Loftus , R. Thilakaratne , and N. Cocks . 2022. “Barriers and Facilitators to Conversation: A Qualitative Exploration of the Experiences of People With Parkinson's and Their Close Communication Partners.” Brain Sciences 12: 944. 35884750 10.3390/brainsci12070944 PMC9321478

[jlcd70084-bib-0039] Yorkston, K. , C. Baylor , and D. Britton . 2017a. “Incorporating the Principles of Self‐Management Into Treatment of Dysarthria Associated With Parkinson's Disease.” Seminars in Speech and Language 38: 210–219. 28618444 10.1055/s-0037-1602840 PMC6583887

[jlcd70084-bib-0040] Yorkston, K. , C. Baylor , and D. Britton . 2017b. “Speech Versus Speaking: The Experiences of People With Parkinson's Disease and Implications for Intervention.” American Journal of Speech‐Language Pathology 26: 561–568. 28654939 10.1044/2017_AJSLP-16-0087 PMC5576965

