# Supplementary material for: The PD COMM Process Evaluation: Describing Interventions and Implementation in a UK Pragmatic Randomised Controlled Trial of Speech and Language Therapy for People With Parkinson's‐Related Dysarthria
Source: Int J Lang Commun Disord. 2025 Jul 10;60(4):e70084. doi: 10.1111/1460-6984.70084 (PMC12244302; doi:10.1111/1460-6984.70084)
Supplement: Supplementary file 1 — Supplementary Appendix 1: PE‐ID coding guide (TIDieR: what materials, what procedures, tailoring) Supplementary Appendix 2: Broad categories of SLT therapy sessions documented in TRF [file JLCD-60-0-s001.docx]

# Appendix 1 PE-ID coding guide (TIDieR: what materials, what procedures, tailoring)

## TIDieR category: What materials

### Technologies

| **Code** | **Definition** |
| --- | --- |
| **Technology** | Physical materials |
| Exercise materials | Include any mention of ‘sheet’ or ‘list’ and any physical materials such as picture (‘picture description’) or passage (‘reading passage’).  May be client’s own materials. |
| Info sheets | Include mention of any ‘sheet’ or ‘information’ that is clear it was reading material. |
| Ref web | Include any referral to information on a website |
| Phone / video call | Any reference to using voicemail, phone, or online communication |
| App | Any reference to ‘app’ or a specific named app for exercises or information |
| AAC (text) | Any reference to low or high tech not in other categories and with aim of being used to assist / modify communication, e.g. pacing board |
| Other (text) | Record in text anything not covered by the above categories |

### Feedback tools

| **Code** | **Definition** |
| --- | --- |
| **Biofeedback (sense)** | Used to increase awareness of normally automatic bodily functions so the person can learn to move and control the body more effectively |
| Visual | e.g. Mirror, Sound Level Meter, app, stopwatch |
| Tactile | e.g. Hand on larynx |
| Resistance | e.g. Tube |
| Auditory | e.g. Voice recording and playback (NB Do not include if listening to differences without biofeedback tool) |

### Numerical measurement

| **Code** | **Definition** |
| --- | --- |
| **Numerical / scaled measurement** | Tool used to provide numerical or scaled measurement (NB Do not include if measurement is non-numerical or unscaled) |
| From object | e.g. app, Sound Level Meter |
| Self-rating | e.g. rating scale |
| Rating by others | e.g. rating scale |

### Named approaches

| **Code** | **Definition** |
| --- | --- |
| **Named approaches** | Drawing on techniques from named approaches which can be referenced |
| Other (text) | Write in any other named approach which meets the definition |

## TIDieR category: What procedures

### Target (speech subsystems)

| **Code** | **Definition** |
| --- | --- |
| **Target (speech subsystems)** | The impairment(s) that therapy activity is targeting, conceptualised around the five speech subsystems (respiration, phonation, articulation, resonance and prosody) and associated behaviours |
| Relax | Reference to relax / relaxation |
| Posture | Reference to posture / sit up straight |
| Breath | Reference to breathing, e.g. diaphragmatic, breath support |
| Voice | Reference to work on phonation / voice – duration, volume, pitch, quality.  NB Includes reference to voice rest and monitoring strain.  NB If unclear that voice is the target (e.g. ‘ahs’ without whether it is voice or artic, say ‘No’ here) |
| Reson | Reference to resonance / soft palate / nasality |
| Artic | Reference to articulation, pronunciation, clarity, precision  NB If unclear that ‘lip exercises’ refers to articulation (e.g. may be swallowing-related) do not record here. |
| Rate | Reference to slowing down, pausing, chunking, pacing |
| Stress | Reference to emphasis, stress, intonation, perhaps with ‘contrastive’ |
| Facial expression | Reference to facial movement or expression |

### Target (other)

| **Code** | **Definition / examples** |
| --- | --- |
| **Target (other)** |  |
| Lang/Cog | Inclusion of explicitly cognitive and / or linguistic elements beyond speech/voice tasks. |
| Insight | Explicit attention to helping people recognise what is different in their communication as a consequence of Parkinson’s, and why this makes it harder for other people to understand them. Includes explicit discussion of impact of cognitive load. Incorporates LSVT ‘calibration’ (retrain sensory perceptions – provide feedback on feeling the effort and make explicit link with that is the effort you need to feel when you are speaking)  NB May include insight around NOT speaking too loudly / voice quality |
| Psychosocial adjustment | Explicit techniques used to address the social, psychological, emotional and / or spiritual needs of people who are adjusting to having, coping and planning for the future with a chronic condition (Parkinson’s) |
| Self-management | Drawing on Yorkston et al (2017) paper on incorporating principles of self-management Parkinson’s dysarthria: helping clients to develop own problem-solving skills; educating to prepare them to make decisions; connecting them to resources; helping them prepare for interactions with other healthcare providers; supporting clients with feasible action plans.  NB Includes agreed maintenance plan. |
| Attention to environment | Strategies to modify the speaking environment, e.g. reduce background noise; equip others with strategy  Also deliberate use of background noise to work against |

### Voice / speech cue(s)

| **Code** | **Definition / examples** |
| --- | --- |
| **Cue(s)** | A word or short phrase which is akin to an aide-memoire for self-cueing  NB Do NOT include if generic rather than a cue to action. |
| Loud | Think loud / loud |
| Strong / be heard / volume | Alternative, multiple or less specific references to volume cues than ‘think LOUD’  NB Can include ‘louder’ if used with another word such as volume. |
| Clear | Clear speech / Be clear |
| Slow | Speak slower / pause |
| Over | Reference to exaggeration / over-doing something or emphasis (e.g. volume, articulation, facial expression, stress) |
| Chunk / pause | Reference to grouping words, e.g. for pacing; incorporates pausing |
| Forward focus | Reference to bringing voice forward |
| Other (text) | Anything not listed above |

### Vocal activities

| **Code** | **Definition** |
| --- | --- |
| **Vocal activities** | The vocal activities done to deliver the exercises |
| NSOME | Non-speech oral motor movement such as blowing, lip stretches (NB Do not include if ONLY for breathing exercises) |
| C/V/CV | Use of sustained phoneme e.g. ah, sh, or CV structures (real word or nonsense) |
| Automatic speech | Counting, alphabet |
| Play | Word play, e.g. tongue twisters, limericks, poems, rhymes, rewording |
| LCU | Manipulation of length and complexity of utterance e.g.: single words, multisyllabic words, phrases, sentences, paragraphs, conversations |
| Funct / everyday phrases | Phrases specifically selected for everyday usage (in LSVT = functional phrases) |
| Interaction | 1. Activities which are focused on interaction with a person other than the therapist (i.e. reciprocity) rather than those which could be done alone NB ‘Conversation’ is not enough as comes under LCU; there needs to be a feedback element. 2. Also covers interaction with the therapist in a real-world / naturalistic setting, e.g. café, waiting room. 3. Also covers evidence of therapist working on ‘off-the-cuff’ conversation / small talk (a big part of LSVT – the therapist needs to attend to loudness in everyday conversation as soon as it drops) |

### Practice structure

| **Code** | **Definition** |
| --- | --- |
| **Practice structure** |  |
| Daily tasks: Yes / Partial / No | Yes = daily tasks as unchanging drills from day 1; Partial = evidence of idea of daily exercises / practice either explicitly, or through repeating drills |
| Hierarch tasks: Yes / Partial / No | Yes = movement across length and complexity of utterance from day 1; Partial = evidence of therapy moving through increasing length and / or complexity of utterance |
| Multi task | Explicit incorporation of dual or more task activities, or a distracting task, sometimes described as deliberate adding of cognitive load |
| Applied Tasks | Equivalent to LSVT carryover tasks; using skills in a specific applied context  (NB Do not include if too generic) |
| HBT review | Explicit that HBT has been reviewed during the session.  This is not necessarily review of prescribed tasks, but of how therapy is impacting on specific situations. |

## TIDieR category: Tailoring

### Personal

| **Code** | **Definition** |
| --- | --- |
| **Personal** |  |
| Interests | Materials or topics |
| Interactions (people / places) | People or places specific to the person  (NB Having functional phrases is not sufficient)  May include discussion / reflection on how has been applying / carrying over, even if these were not prescribed tasks. |
| Goals | To specific outcomes or goals identified by participant |
| Health | Adjustments made by therapist to account for health or other contextual factors e.g. bereavement;  Health interfering with ability to do home practice or commit to therapy |

### Interpersonal

| **Code** | **Definition** |
| --- | --- |
| **Interpersonal** |  |
| Model | Indicated by words such as model; demonstrate; facilitate; shape (also relevant to Titrate); or participant comment on loudness of therapist’s voice |
| Encourage | Indicated by words such as encourage; praise; enthusing patient; coaching; - but also by suggestions to be more proactive in communication, or to do things differently |
| Advise / educate | Indicated by words such as advised, educated, reassured.  Note where ‘educate’ is more than giving information as examples of good practice. |
| Titrate | Indicated by words such as prompt; re-shape / shaping; cue; remind; feedback; pushed; used visual imagery; but also went through / practised / revised / ran through / reinforce  Also indicated by any adaptation made by therapist to make task more easy or challenging, perhaps referred to as step up/down. |
| Involve others | Active involvement of others in therapy, directly or indirectly (through tasks which ask participant to ask for and report feedback) |
| Other (text) |  |

# Appendix 2 Broad categories of SLT therapy sessions documented in TRF

- Assessment and review
- Goal setting
- Information provision (to the person or the caregiver)
- Impairment exercises
- Compensatory strategies
- Alternative and augmentative communication strategies
- Generalisation activities
- Training of caregivers
- LSVT
- Indirect contact / liaison
- Other
